# Owl Monkeys (*Aotus nigriceps* and *A*. *infulatus*) Follow Routes Instead of Food-Related Cues during Foraging in Captivity

**DOI:** 10.1371/journal.pone.0115188

**Published:** 2014-12-17

**Authors:** Renata Souza da Costa, Júlio César Bicca-Marques

**Affiliations:** Pontifícia Universidade Católica do Rio Grande do Sul, Faculdade de Biociências, Porto Alegre, Rio Grande do Sul, Brasil; CNR, Italy

## Abstract

Foraging at night imposes different challenges from those faced during daylight, including the reliability of sensory cues. Owl monkeys (*Aotus* spp.) are ideal models among anthropoids to study the information used during foraging at low light levels because they are unique by having a nocturnal lifestyle. Six *Aotus nigriceps* and four *A. infulatus* individuals distributed into five enclosures were studied for testing their ability to rely on olfactory, visual, auditory, or spatial and quantitative information for locating food rewards and for evaluating the use of routes to navigate among five visually similar artificial feeding boxes mounted in each enclosure. During most experiments only a single box was baited with a food reward in each session. The baited box changed randomly throughout the experiment. In the spatial and quantitative information experiment there were two baited boxes varying in the amount of food provided. These baited boxes remained the same throughout the experiment. A total of 45 sessions (three sessions per night during 15 consecutive nights) per enclosure was conducted in each experiment. Only one female showed a performance suggestive of learning of the usefulness of sight to locate the food reward in the visual information experiment. Subjects showed a chance performance in the remaining experiments. All owl monkeys showed a preference for one box or a subset of boxes to inspect upon the beginning of each experimental session and consistently followed individual routes among feeding boxes.

## Introduction

The efficient exploitation of food resources in different ecological contexts depends on the cognitive abilities of the forager [1], which include perception (e.g. vision, olfaction and audition), spatial memory (location of an object in space), and the ability to use associative cues (relationship between food sources and objects or environmental traits) and quantitative information (differences in the amount of food) in the decision-making process [2], [3]. The ability to apply and to integrate the knowledge acquired in previous situations increases foraging success. The forager may, for example, integrate spatial, visual and olfactory information to estimate the quantity and/or quality of food available and develop exploitation strategies [2], [4], [5].

The activity pattern (diurnal, nocturnal or cathemeral) also impacts the efficiency of using certain ecological information [2]. While a diurnal animal can rely on visual acuity to locate food, a nocturnal animal has to rely on acute olfaction and/or more sensitive auditory perception to compensate for the reduced vision accuracy in a low-light environment [6], [7].

The way animals use ecological information during foraging can be studied in a natural environment, in semi-captivity or in captivity [8], [9]. Despite being a spatially restricted environment, when cognitive studies are conducted in captivity, there can be greater control of environmental variables that may affect behavior [10]. Furthermore, captivity may be particularly useful for the study of species that are not easily spotted in nature, such as nocturnal ones. This is the case of the owl monkeys [11].

The owl monkeys (*Aotus* spp.) are the only anthropoid primates with a nocturnal lifestyle [12] (some populations exhibit cathemeral activity pattern [13]). Among their adaptations for activity in a low-light environment are enlarged eyes, a retina with only one type of cone (which results in monochrome vision) and changes in the lens and iris diaphragm [14], [15], and a relatively larger olfactory bulb compared to other New World primates [16], [17].

Experiments with free-ranging and semi-captive platyrrhines concluded that olfactory information is more salient to the owl monkeys than to the diurnal species, while the opposite is observed in relation to visual information [2], [16]. On the other hand, the diurnal and nocturnal monkeys exhibit similar efficiency in the use of spatial information (predictable food location; [2]). According to Bolen and Green [16], the owl monkeys follow a consistent route during foraging in semi-captivity. Wright [17], [18] and Castaño et al. [19] believe that free-ranging owl monkeys identify the routes by scent marking and visual cues. Wright [17] also suggests that the sense of hearing plays an important role in foraging for invertebrates in the natural environment [17], but this ability is yet to be evaluated experimentally. It is known, however, that the auditory sensitivity of owl monkeys is similar to that of the diurnal squirrel monkeys (*Saimiri* spp.) [20].

The current study aimed to test the ability of captive owl monkeys (*Aotus nigriceps* and *A. infulatus*) to use visual, auditory, olfactory, spatial, and quantitative cues in nocturnal foraging decision-making and to evaluate whether they follow consistent routes between feeding locations as a foraging strategy.

## Material and Methods

### Ethical note

**Figure 1:**
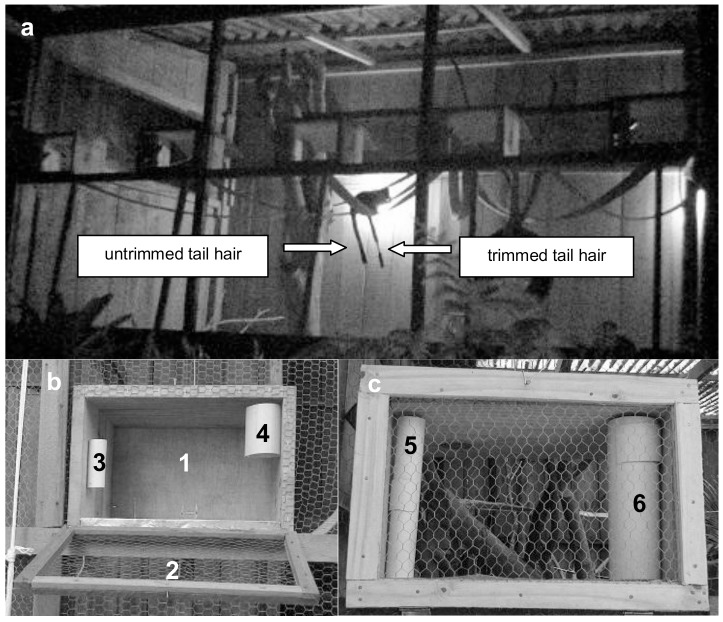
Experimental setting. (a) Enclosure inhabited by group 4 (note the tail hair trimming in female F4 and the untrimmed tail hair of male M4); (b) feeding box with closed inner door (1), outer door open (2), support where the smell-tube was attached (3) and the support where the food-tube was attached (4); (c) feeding box with the outer door closed and the inner one open and with the smell-tube (5) and the food-tube (6) installed.

The research was approved by the Ethical Committee of the Pontifical Catholic University of Rio Grande do Sul (registration # 11/00259) and complies with the Brazilian environmental legislation. A small portion of the females' tail hair of groups 1, 2 and 4 was trimmed so that the individuals of each enclosure could be identified (Fig. 1a). For group 5, the tip of F52's tail hair was trimmed while the male's was trimmed on the posterior end. Tail trimming was conducted when the monkeys were captured with a net and chemically restrained with Ketamin S+ at a dose of 10 mg/kg administered intramuscularly for standard procedures of the facility. No analgesic was used because hair trimming causes no pain. Handling was performed solely by the veterinarian Moira Ansolch da Silva. None study subject was sacrificed during or after completion of this observational study. This research adheres to the ARRIVE Guidelines for reporting animal research (see ARRIVE checklist in S1 Checklist).

### Study site and subjects

This study was conducted at the Criadouro Conservacionista de Animais Silvestres Arca de Noé (29°31'43.53''S, 51°04'13.26''W), Morro Reuter, state of Rio Grande do Sul, Brazil, with 10 adult owl monkeys (six *A. nigriceps* (ng) and four *Aotus infulatus* (if); five males (M) and five females (F)). The animals were divided into three pairs (group 1 (ng): F1 and M1; group 2: F2 (ng) and M2 (if), group 4 (if): F4 and M4), a lone male (group 3: M3 (if)) and a trio (group 5 (ng): F51, F52 and M5) in five enclosures with areas ranging from 42 to 105 m^3^ (S1 Table). The females F2 and F52 are daughters of F51 and M5. Study groups were formed by the staff of the facility before the animals were identified at the species level.

Enclosures were naturalized with soil substrate and shrubs and enriched with wood perches and firefighter hoses (see below). Each enclosure had a nest-box (30 cm wide ×30 cm long ×55 cm high, groups 1 to 4; 52×30×40 cm, group 5) heated by a 40 W lamp that was used as a shelter at any time and for sleeping during daytime. Fresh water was available *ad libitum* from a drinking fountain in each enclosure. The animals were fed once a day at night with a mix of fruit, leaves, eggs, meat, and monkey and dog chow.

### Experimental setting

Five visually similar feeding boxes made of wood and measuring 30×40 cm at the base and 30 cm high were installed in each enclosure (Fig. 1a). All boxes were fixed to the enclosure's fence at *ca*. 1.5 m above the ground. The distance between adjacent feeding boxes varied from 30 to 40 cm depending on the length of the enclosure's front. Each box had a food-tube and a smell-tube (see below), an outward opening screen door and an inward opening wooden door (Figs. 1b and 1c). The outward facing door gave access to the inner part of the boxes so that the experimental conditions could be prepared and the behavior of the animals inside could be observed. The inner door prevented the animals from observing the preparation of the experiment and only allowed access to the box during the experimental session. These doors were triggered simultaneously from the outside of the enclosure by a pulley system. A security lock on the inner door prevented the animals from escaping when the outer door was open. Besides making it possible for the experiment to be conducted, the boxes increased the cost of foraging by reducing the ability of the animals to monitor the behavior of their cage mate(s) while they were inspecting a box. Therefore, the boxes reduced the feasibility of using social information in foraging decision-making.

Firefighter hoses were installed in the enclosures to provide similar access pathways to all boxes. The hoses stretched from the center of each box and converged at a single point at the back of the enclosure. Due to the owl monkeys' low sensitivity to red light [14], two red 15 W lamps were installed at the back of the enclosure, similar to the one used by Bicca-Marques and Garber [2] in an experimental field study. This was done to improve the researcher's ability to see the boxes and animals during the night.

Bananas were used as a reward because they were easy to get throughout the study and they are consumed by the animals in the facility. During the study, banana was not offered to the animals in their regular meal. It was used only during the experimental sessions in order to keep the animals interested in the reward. In the morning following each night of data collection, the remains of food were removed from the enclosure so that the monkeys would not eat before the three experimental sessions at night. Depending on the experimental protocol, real bananas or plastic bananas (similar in size, shape and color to real bananas) were used as bait. After the preparation of the five feeding boxes (distribution of banana slices on the food- and smell-tubes; see below) of each experimental session and the closing of the outer doors, the inner doors were opened simultaneously in order to provide the animals with the same opportunity to choose the boxes.

The rewards were placed inside a PVC food-tube (75 mm in diameter and 25 cm in height) in order to increase the cost of foraging. A hole with a diameter of 5 cm was opened in the middle of the food-tube so that the animals could explore its interior. The size of the tube and hole were set in a pilot study so that the animals could put their hands and part of their arms in the interior, but they could not see the content from outside the feeding box.

In order to homogenize the banana odor released by the reward boxes during the experiments (except for the olfactory one, see below), a PVC smell-tube (40 mm diameter) was fixed inside the box. This tube prevented the animals from seeing the contents (banana pieces), but small holes in its upper part released the odor. Each box's smell-tube had the same number of holes so that the same odor was released. The smell-tube of the reward box remained empty, because the odor was released by the food-tube. The smell- and food-tubes were cleaned every day with mild soap and water. The base of the box was lined with clear plastic and cleaned with a damp cloth before each session in order to eliminate possible odors from the previous session.

### Experiments

Four experiments tested the monkeys' ability to use the following information: (1) olfactory (odor present only in the reward box), (2) visual (differences between real and fake bananas), (3) auditory (sound present in the reward box), and (4) spatial (constant location of the reward) + quantitative (differences in the amounts of rewards available in predictable locations) in foraging decision-making, i.e. choosing which feeding boxes to visit in search of the food reward. Prior to these four experiments, a control experiment (no sensory, spatial or quantitative information available) was conducted to assess whether the animals were using some information not controlled by the experimental protocol to find the food rewards (Table 1). In the sound experiment, a clock that emitted a 25 dB noise ("tick-tack") was used. Its intensity was measured with the Sound Level Meter app available at https://play.google.com (Smart Tools Co., version 1.5.10) and is within the auditory sensitivity of the owl monkeys [20].

**Table 1:** Description of the experiments and time they were conducted.

EXPERIMENT	CONDITIONS
**Control** (1^st^ to 15 April 2012)	Visual cues absent: RB contains hidden banana slices inside the food-tube
	Olfactory cues minimized: NRBs contain inaccessible banana slices inside the food-tube
	Spatial information unavailable: the location of RB changed randomly in each session
**Olfactory cues** (28 April to 12 May 2012)	Visual cues absent: RB contains hidden banana slices inside the food-tube
	Olfactory cues available: NRBs contain empty food-tube and smell-tube
	Spatial information unavailable: the location of RB changed randomly in each session
**Visual cues** (26 May to 9 June 2012)	Visual cues present: RB contains 7 cm of banana out of the food-tube; NRBs contain 7 cm of plastic banana out of the food-tube
	Olfactory cues minimized: RB contains an empty smell-tube; NRBs contain 7 cm of banana inside the smell-tube
	Spatial information unavailable: the location of RB changed randomly in each session
**Auditory cues** (23 June to 7 July 2012)	Visual cues absent: RB contains hidden banana slices inside the food-tube
	Olfactory cues minimized: NRBs contain inaccessible banana slices inside the food-tube
	Spatial information unavailable: the location of RB changed randomly in each session
	Auditory cues present: RB emits a 25 dB tick-tack
**Spatial and quantitative information** (21 July to 4 August 2012)	Visual cues absent: RBs contain hidden banana slices inside the food-tube
	Quantity information available: RB3 contains three half banana slices inside the food-tube; RB1 contains only one half banana slice inside the food-tube
	Olfactory cues minimized: RB3 contains an empty smell-tube; RB1 contains two half banana slices inside the smell-tube; NRBs contain three half banana slices inside the smell-tube
	Spatial information available: the location of RBs remained constant throughout the experiment

In four experiments (not the spatial + quantitative), only one box, chosen using a table of random numbers (available in [21]), received accessible food rewards (reward box, RB) in each experimental session of all enclosures, while the other boxes had empty food-tubes or food-tubes baited with inaccessible banana slices (non-reward boxes, NRB). Hence, there was a 20% probability of individuals randomly choosing the RB. In the spatial + quantitative experiment there were two RBs (with different amounts of reward) and a 40% probability (2/5) of visiting a RB at random.

The amount of food given to the animals was standardized on a 1.5 cm thick slice of banana, except in the visual experiment and the spatial + quantitative experiment. The slice was offered divided in half, so that more than one individual could feed in a RB. The amount of banana in the smell-tube was always equal to that of the food-tube, in order to equalize the odor released by each box, except in the olfactory experiment (Table 1). The diameter of the banana slices varied from 2 to 4 cm (mean ± sd = 2.9±0.3 cm, N = 1,350 slices).

### Data collection

Each experiment lasted 15 consecutive nights. Each night, three experimental sessions were conducted per enclosure (15 nights ×3 sessions/night  = 45 sessions/group per experiment) resulting in 225 sessions with each and every group throughout the study. Each experimental session lasted 5 min., allowing enough time to inspect all feeding boxes according to a pilot study conducted between March 2011 and March 2012. The number of sessions in each experiment was adapted from the protocol used by Bicca-Marques [22]; Bicca-Marques and Garber [2], [3], [23]. There was an interval of 13 consecutive days between experiments in order to extinguish any learning from the previous experiment and to reduce its influence on the animals' performance in the following experiment. No rewards were placed in the smell- and food-tubes and the inner doors of the boxes were kept closed, so that the boxes could not be inspected by the animals during the interval.

Data collection began at night (between 06:00 PM and 08:00 PM), when the animals started their activities. The starting time followed the photoperiod changes. The first session started every evening at a different enclosure based on a rotation system. For example, the first data collection night of an experiment started at enclosure 1, followed by enclosures 2, 3, 4 and 5 in that order. On the second night, the collection started at enclosure 2 followed by enclosures 3, 4, 5 and 1, and so on. The three sessions of each data collection night (see below) followed the same order. This rotation was performed to homogenize data collection within all groups in terms of time.

Due to the speed in which animals visited and inspected the feeding boxes, all experimental sessions were filmed with a Sony Camcorder DCR - SR300 equipped with infra-red for later analysis, totaling 5,625 minutes (or 93h45min) of recording in 1,125 videos. The following data were recorded using the "all occurrences" sampling method [24]: subject identity, number of the box visited or inspected (see below), latency of arrival on the first box chosen by each subject, and occurrence of feeding. The choice was considered successful when the individual entered the RB. On the other hand, the choice was considered unsuccessful when the subject entered a NRB. During each session, the first entry of any individual in each box was called "inspection". Thus, each group could carry out up to five inspections in each experimental session, i.e. one “inspection” per box. When the same individual returned to the box already inspected or another individual entered the same box, even if for the first time, this event was called "visit". The time it took each individual to inspect or visit its first box (latency) was recorded in seconds.

### Data analysis

The chi-square test was used to determine if the frequency with which each box was selected to receive the reward in each experiment did not differ from what was expected by chance. This analysis aimed at evaluating the possible influence of non-intentional spatial information (frequent location of food in a particular box) on the animals' performance in the experiments (except spatial + quantitative). The frequency that each box was a RB throughout the experiments with no spatial information available ranged from 9 to 38% (mean ± sd = 20±7, N = 20).

The performance of individuals in each experiment was evaluated taking into account the sum of successful choices in their first inspection, i.e. when the individual went straight to a RB not yet visited by another individual at the beginning of the session. When the first choice of an individual in a session was a visit to a RB already inspected by another individual, this session was excluded from the analysis of its performance.

The binomial non-parametric one-tailed test was used in order to determine if the performance was higher than expected by chance of (a) 20% (1/5) for selecting the RB (i.e. that by chance the animals would inspect the RB as their first choice in up to nine of 45 sessions of each experiment) in the control, olfactory, auditory and visual information experiments [3], (b) 40% (2/5) in the spatial experiment (up to 18 successful choices) and (c) 50% (1/2) for choosing the RB with the highest amount of food in the quantitative experiment (up to 22 successful choices). In order to assess whether the individual performance was improving with experience throughout each experiment, we analyzed the distribution of its successful inspections (when it went straight into a RB in the session) over the 45 sessions of the experiment by means of a binomial logistic regression. The chi-square test was used to assess if the frequency in which each box was the first choice of every individual in each experiment deviated significantly from the expected 20% (1/5, i.e. randomly every box would be the first choice in nine sessions) to verify whether the owl monkeys had a preference for one box or a set of boxes, or if the first choice was random throughout each experiment.

In order to test whether the subjects used repeated foraging routes between the feeding boxes during the study, a matrix with all transitions between the five feeding boxes for each individual in each experiment was drawn, as performed by Bicca-Marques and Nunes [25]. The total number of transitions made by each individual in each experiment was divided by the value of possible transitions (20) to give the expected value for each matrix cell (each transition) considering a homogeneous distribution. The chi-square test was used to verify if the number of observed transitions differed from the expected value. All tests assumed a significance level of 5% and were performed with the BioEstat 5.0 software [26].

## Results

The owl monkeys made 5,397 inspections and 16,965 visits ( = 22,362 inspections + visits) throughout the study or an average of 4.80 inspections and 15.1 visits per session. As soon as the access to the boxes was released, all subjects began the search for food. A total of 1,565 feeding events (mean ± sd  = 157±76 per subject) were recorded.

Control experiment: the frequency with which each individual selected the RB for their first inspection during each session (i.e. went straight to the RB) varied from 4 to 12 (Table 2). No subject performed above chance level. While M4 had a more evenly distributed first choice among the boxes, other individuals concentrated their first choices on one or two boxes only (Table 3).

**Table 2:** Individuals' performance on the first choice of each session during each experiment showing the amount of successful choices (and their respective %), result of the binomial test (Z and P) for the values >9 (all experiments, except the spatial + quantitative) and >18 (spatial + quantitative experiment) and average, median, minimum and maximum latency (in seconds) between the start of each session and the inspection of the first box.

Experiment	F1	M1	F2	M2	M3	F4	M4	F51	F52	M5
**Control**										
Successful	12 (26.7%)	7	8	7	9	5	4	6	11 (26.1%)	5
Z	1.12	.	.	.	.	.	.	.	1.00	.
P	.13	.	.	.	.	.	.	.	.16	.
Latency										
Minimum	1	1	1	1	1	1	1	1	1	1
Maximum	4	7	17	37	22	7	45	79	119	141
Median	1	1	3	2	4	1	8	5	9	8
Average	1.29	1.56	3.84	3.93	5.69	1.40	11.5	9.78	11.5	13.2
**Olfactory**										
Successful	10 (22.2%)	6	6	12 (26.7%)	**14 (31.1%)**	10 (22.2%)	5	6	5	10 (22.7%)
Z	0.37	.	.	1.12	**1.86**	0.37	.	.	.	0.45
P	.35	.	.	.13	**.031**	.35	.	.	.	.32
Latency										
Minimum	1	1	1	1	1	1	1	1	3	1
Maximum	2	3	15	9	67	62	188	32	49	32
Median	1	1	2	1	5	1	4	5	9	7
Average	1.04	1.18	2.82	1.53	7.33	3.91	12.7	6.48	11.7	7.11
**Visual**										
Successful	**16 (35.6%)**	7	6	10 (22.2%)	11 (24.4%)	9	8	12 (26.7%)	4	10 (23.8%)
Z	**2.61**	.	.	0.37	0.74	.	.	1.21	.	0.62
P	**.045**	.	.	.35	.23	.	.	.11	.	.27
Latency										
Minimum	1	1	1	1	1	1	1	2	4	1
Maximum	15	16	23	9	252	232	73	96	241	83
Median	1	1	2	1	5	4	3	7	10	3
Average	1.89	1.51	2.56	1.60	22.5	14.6	10.0	16.4	29.5	12.3
**Auditory**										
Successful	8	11	9	11 (24.4%)	13 (28.8%)	8	8	12 (26.7%)	6	8
Z	.		.	0.74	1.52	.	.	1.12	.	.
P	.		.	.23	.064	.	.	.13	.	.
Latency										
Minimum	1	1	1	1	1	1	1	1	2	1
Maximum	2	23	10	34	108	4	6	71	104	114
Median	1	1	1	1	3	1	2	3	7	1
Average	1.04	1.89	2.02	1.93	6.64	1.13	1.93	6.56	11.9	9.00
**Spatial**										
Successful	18	0	11	**36 (80.0%)**	1	22 (50.0%)	9	3	9	21 (48.8%)
Z	.	.	.	**5.48**	.	1.22	.	.	.	1.18
P	.	.	.	**<.0001**	.	.11	.	.	.	.12
Latency										
Minimum	1	1	1	1	1	1	1	1	1	1
Maximum	1	3	19	236	26	62	63	80	64	73
Median	1	1	1	47	4	1	1	4	4	1
Average	1.00	1.04	1.80	52.8	5.09	7.29	5.84	7.98	7.60	7.18

Significant results are in bold.

**Table 3:** Frequency of box choice for the first inspection in each session (n and respective %) and the result of the chi-square test to assess the preference for certain boxes (χ^2^ and P; d.f.  =  4 for all) is also shown.

	F1	M1	F2	M2	M3	F4	M4	F51	F52	M5
Box	n (%)	n (%)	n (%)	n (%)	n (%)	n (%)	n (%)	n (%)	n (%)	n (%)
**Control**										
1	3 (6.7)	38 (86.4)	.	6 (13.6)	27 (60.0)	.	6 (14.2)	29 (65.9)	17 (40.5)	2 (4.4)
2	31 (68.9)	6 (13.6)	2 (4.5)	3 (6.8)	16 (35.6)	2 (4.4)	12 (28.6)	2 (4.5)	3 (7.1)	3 (6.7)
3	7 (15.6)	.	13 (29.5)	1 (2.3)	1 (2.2)	7 (15.6)	4 (9.5)	.	11 (26.2)	9 (20.0)
4	4 (8.9)	.	15 (34.1)	30 (68.2)	1 (2.2)	28 (62.2)	9 (21.4)	1 (2.3)	7 (16.7)	23 (51.1)
5	.	.	14 (31.8)	4 (9.1)	.	8 (17.8)	11 (26.2)	12 (27.3)	4 (9.5)	8 (17.8)
χ^2^	70.0	124	23.5	65.3	64.7	55.1	4.89	68.5	13.1	15.6
P	**<.0001**	**<.0001**	**<.0001**	**<.0001**	**<.0001**	**<.0001**	.30	**<.0001**	**.011**	**.004**
**Olfactory**										
1	.	43 (95.6)	.	.	14 (31.1)	3 (6.7)	6 (13.6)	27 (61.4)	5 (11.9)	3 (6.8)
2	39 (86.7)	1 (2.2)	2 (4.5)	.	24 (53.3)	14 (31.1)	12 (27.3)	9 (20.5)	19 (45.2)	3 (6.8)
3	6 (13.3)	.	25 (56.8)	3 (6.7)	5 (11.1)	11 (24.4)	8 (18.2)	4 (9.1)	15 (35.7)	13 (29.5)
4	.	1 (2.2)	15 (34.1)	20 (44.4)	2 (4.4)	14 (31.1)	13 (29.5)	2 (4.5)	1 (2.4)	18 (40.9)
5	.	.	2 (4.5)	22 (48.9)	.	3 (6.7)	5 (11.4)	2 (4.5)	2 (4.8)	7 (15.9)
χ^2^	128	161	53.5	54.2	44.0	14.0	5.77	50.8	31.3	19.6
P	**<.0001**	**<.0001**	**<.0001**	**<.0001**	**<.0001**	**.007**	.22	**<.0001**	**<.0001**	**.0006**
**Visual**										
1	1 (2.2)	45 (100)	.	2 (4.4)	22 (48.9)	7 (15.6)	11 (24.4)	23 (52.3)	6 (15.4)	1 (2.4)
2	18 (40.0)	.	.	.	16 (35.6)	10 (22.2)	15 (33.3)	9 (20.5)	20 (51.3)	2 (4.8)
3	23 (51.1)	.	13 (29.5)	.	4 (8.9)	11 (24.4)	7 (15.6)	6 (13.6)	11 (28.2)	14 (33.3)
4	2 (4.4)	.	31 (70.5)	9 (20.0)	3 (6.7)	14 (31.1)	9 (20.0)	3 (6.8)	1 (2.6)	19 (45.2)
5	1 (2.2)	.	.	34 (75.6)	.	3 (6.7)	3 (6.7)	3 (6.8)	1 (2.6)	6 (14.3)
χ^2^	50.4	180	84.4	92.9	40.0	7.78	8.89	31.4	32.7	29.2
P	**<.0001**	**<.0001**	**<.0001**	**<.0001**	**<.0001**	.1001	.0639	**<.0001**	**<.0001**	**<.0001**
**Auditory**										
1	.	44 (100)	3 (6.7)	3 (6.7)	33 (73.3)	.	10 (22.2)	37 (82.2)	4 (10.3)	1 (2.3)
2	43 (95.6)	.	.	.	8 (17.8)	4 (8.9)	18 (40.0)	6 (13.3)	21 (53.8)	5 (11.6)
3	2 (4.4)	.	15 (33.3)	.	2 (4.4)	13 (28.9)	4 (8.9)	1 (2.2)	11 (28.2)	20 (46.5)
4	.	.	27 (60.0)	11 (24.4)	2 (4.4)	13 (28.9)	12 (26.7)	.	3 (7.7)	17 (39.5)
5	.	.	.	31 (68.9)	.	15 (33.3)	1 (2.2)	1 (2.2)	.	.
χ^2^	161	176	62.0	76.2	84.0	19.3	20.0	111	36.2	40.1
P	**<.0001**	**<.0001**	**<.0001**	**<.0001**	**<.0001**	**.0007**	**.0005**	**<.0001**	**<.0001**	**<.0001**
**Spatial +**										
**quantity**										
1	.	45 (100)	.	1 (2.2)	29 (64.4)	3 (6.7)	3 (6.8)	33 (73.3)	12 (36.4)	3 (7.0)
2	27 (60.0)	.	.	.	14 (31.1)	9 (20.0)	12 (27.3)	6 (13.3)	10 (30.3)	2 (4.7)
3	18 (40.0)	.	10 (22.2)	1 (2.2)	1 (2.2)	11 (24.4)	4 (9.1)	1 (2.2)	9 (27.3)	19 (44.2)
4	.	.	34 (75.6)	8 (17.8)	1 (2.2)	11 (24.4)	20 (45.5)	3 (6.7)	2 (6.1)	17 (39.5)
5	.	.	1 (2.2)	35 (77.8)	.	11 (24.4)	5 (11.1)	2 (4.4)	.	2 (4.7)
χ^2^	72.0	180	94.7	98.4	70.4	5.33	23.5	81.5	16.8	34.5
P	**<.0001**	**<.0001**	**<.0001**	**<.0001**	**<.0001**	**.2548**	**.0001**	**<.0001**	**.0021**	**<.0001**

Significant results are in bold.

**Figure 2:**
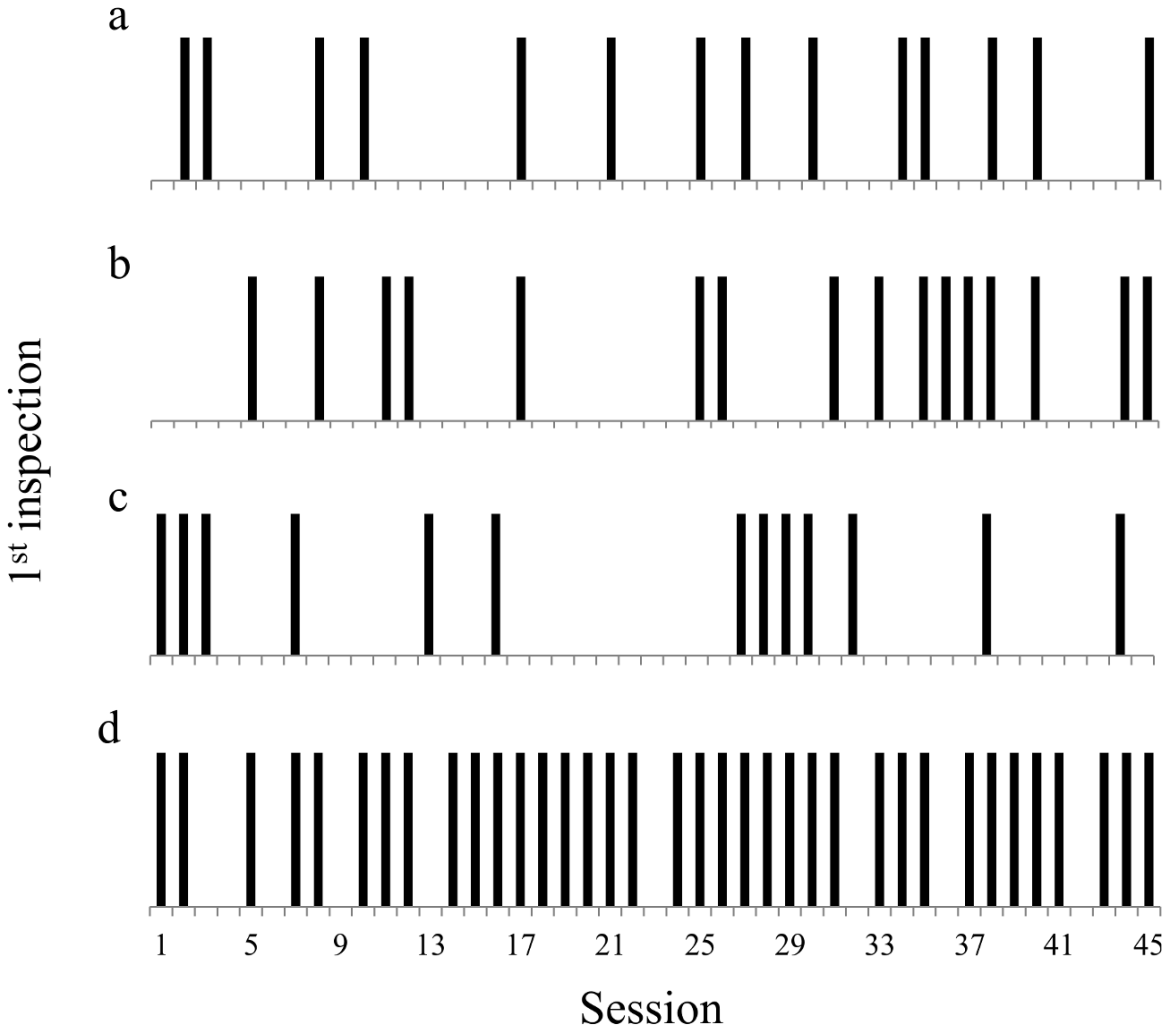
Individual performance on the first inspection. Distribution of successful (black bar) and unsuccessful (no bar) first inspections over the 45 sessions of an experiment. A successful inspection was scored when the study subject went straight into a reward box in the session. (a) M3, olfactory; (b) F1, visual; (c) M3, auditory; (d) M2, spatial.

Olfactory cues experiment: the lone male M3 was the only individual to have a number of successful choices significantly above chance (14 successful choices  = 31.1%) (Table 2). However, his significant performance seems to have resulted from his preference for boxes 1 and 2 (84.4% of his choices), which were RB in 37.8% of sessions. This preference was also observed in the control experiment. This hypothesis is supported by the analysis of the distribution of M3's first correct inspections during the experiment. If M3 was learning (or learned) to use olfactory information, one would expect that his performance would improve during the course of the experiment, a pattern that was not observed (Z = 0.32, P = .74, Fig. 2a). Again, only M4 made his first choices in a more evenly distributed pattern among the boxes (Table 3).

Visual cues experiment: only F1 reached a number of successful choices significantly above chance (16 successful choices  = 35.6%, Table 2). However, as 91.1% of F1's first successful choices concentrated on boxes 2 and 3, which were RB in 51.1% of the sessions throughout the experiment, it is not possible to rule out the hypothesis that her performance is related to the preference for these boxes, especially box 2. Analyzing her first choices throughout the experiment, an increase in the concentration of successful choices in the last third of the sessions suggests a learning curve, although the trend was not significant (Z = 1.75, P = .079, Fig. 2b). F4 and M4 showed a more homogeneous distribution of first choice among the boxes in this experiment, while the remaining individuals continued to show a preference for one or two boxes only (Table 3).

Auditory cues experiment: no individual showed a significant performance in the use of auditory information and all of them showed an uneven choice of boxes for the first inspection (Table 3). The lone male got 13 successful choices, approaching a significant performance (Z = 15.2, P = .064). However, the distribution of his successful choices throughout the experiment was apparently random (Z = 0.73, P = .46, Fig. 2c).

Spatial and quantitative cues experiment: only M2 presented a performance significantly above 40% with 36 successful choices (80.0%, Table 2). However, M2 already showed preference for box 5 (RB) in the previous three experiments (olfactory, 48.9%; visual, 75.6%; auditory, 68.9%), which casts doubt on the cause of his performance (Table 3). A learning curve is also not supported by the analysis of the temporal distribution of his successful choices (Z = 1.10, P = .27, Fig. 2d). Therefore, no individual showed evidence of having used the spatial information above chance level. Consequently, the difference in the amount of reward between the two RBs was also not relevant information in foraging decision-making for the owl monkeys. As in previous experiments, most subjects were biased in choosing the first box to visit. Only F4 showed an even choice of boxes for her first inspection during the sessions (Table 3).

**Figure 3:**
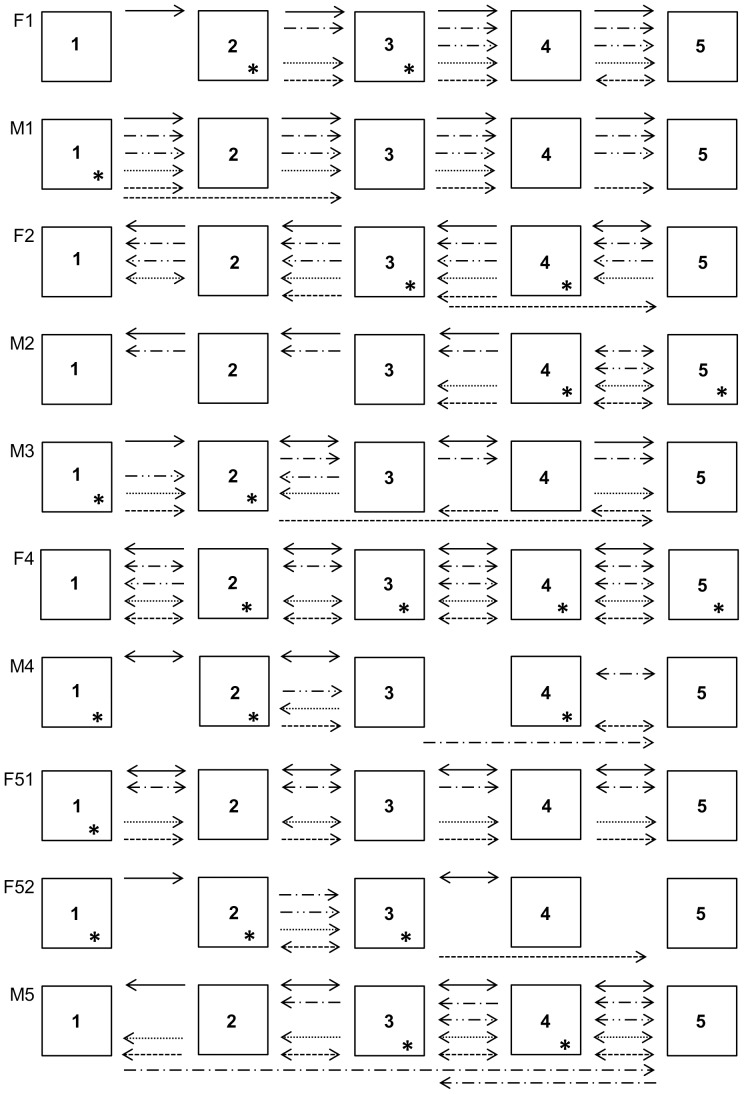
Foraging routes followed by the study subjects in each experiment. Transitions between feeding boxes (arrows) performed with a frequency higher than expected by chance in each experiment. The arrow points from the source box to the target box. The double-headed arrows indicate that the transition was significant in both directions. Asterisks identify the boxes that were selected for first inspection at a frequency higher than expected by chance throughout the study. ___  =  control, -.-  =  olfactory, -..-  =  visual, …  =  auditory, ---  =  spatial + quantitative.

Routes of foraging: all subjects showed a significant difference in the distribution of the transitions between feeding boxes in all experiments. In general, the owl monkeys visited adjacent boxes in sequence. This preference for certain transitions resulted in repeated use of the same routes of foraging in several experiments, regardless of the available sensory cues and the location of rewards in the previous section (Fig. 3). The joint analysis of the transitions made by each individual throughout the study reinforces the owl monkeys' behavior in visiting adjacent boxes in sequence: χ^2^_F1_ = 2158, χ^2^_M1_ = 1268, χ^2^_F2_ = 2125, χ^2^_M2_ = 1383, χ^2^_M3_ = 867, χ^2^_F4_ = 2407, χ^2^_M4_ = 341, χ^2^_F51_ = 1537, χ^2^_F52_ = 346, and χ^2^_M5_ = 2067 (all d.f. = 19, P<.0001).

## Discussion

The owl monkeys showed a performance at random in using the ecological information (olfactory, visual, auditory, spatial + quantitative) available in each experiment to locate the food rewards. Only the female F1's performance in the last third of the visual experiment suggests that she was learning how to use such information to find the banana in the reward box. Therefore, it was not possible to confirm the skills described by Bicca-Marques and Garber [2] for *Aotus nigriceps* (spatial, visual and olfactory) and Bolen and Green [16] for *Aotus nancymaae* (olfactory). The random performance during the control experiment indicates that the owl monkeys did not use information that was not controlled by the experimental protocol to locate the food rewards.

Kamil [27] warns that negative results in cognitive experiments should be interpreted with caution because they may not properly assess the skills used by animals under normal foraging conditions (see also [28], [29]). This limitation may be particularly relevant for studies on captive animals. According to Carlstead [30], it is possible that the performance of animals bred in captivity, subjected to permanent routines of food availability and feeding schedules, is related to the lack of opportunities for them to learn that their behavior can modify the environment and the outcomes of foraging. Considering that the experimental design appropriately reached the threshold for sensory detection (*e.g.*, odor concentration and sound frequency) by owl monkeys, the duration of the experiment is another important aspect that may have influenced the speed or efficacy of the translation from detection to cue recognition and behavioral action (see [31]). Although the number of sessions per experiment in the present study was greater than that successfully employed by Bicca-Marques and Garber [2] for wild owl monkeys (visual: 29 sessions; spatial: 22; olfactory: 12), captive study subjects might, for example, require more time (longer experiments or a greater number of sessions/daily repetitions) to identify the information that is reliably associated to the location of food rewards.

The foraging scale is another factor that can affect the outcome of cognitive tests, since it influences the reliability of ecological information [2], [5], [32]. Foraging can occur on a large-scale (search for food between feeding patches), small-scale (search for food within a feeding patch) or micro-scale (selection of food that is at close range to the forager) [2], [5], [9]. Spatial information, for example, may be more reliable than sensory cues (olfactory, visual and auditory) during large-scale foraging, but is negligible in micro-scale foraging [5], [9], [33]. Although the experimental design has aimed to simulate a small-scale foraging situation, in which olfactory, auditory, visual and spatial + quantitative information would be useful, the close proximity of the feeding boxes may have hindered their coding as independent entities by the animals. This skill would be particularly required for a performance above chance in the spatial experiment.

The small amount of banana offered as a reward in each session, which aimed to keep the monkeys interested in inspecting the boxes in three daily sessions and, at the same time, to reduce the impact of the experimental protocol on the animals' diet (remember that they were exposed to the experiment when fasting soon after waking up on the nest boxes and only received the meal offered by the facility after the three sessions), may not have been stimulating enough to elicit their learning. There may also not have been enough odor released to be perceived and/or used in owl monkey foraging decision-making. However, the low latency (few seconds) between the start of each session and the inspection of the first box by most individuals and the number of inspections and visits done throughout the study indicate that the owl monkeys were interested in the search for food rewards.

The proximity between the boxes also allowed a rapid exploitation of all boxes in sequence with a low or negligible energy cost. While this strategy may reflect an attempt to win the intragroup competition for the small amount of banana available, the behavior of the lone male does not support this hypothesis. Similar to the individuals who shared the enclosure with a mate, the lone male also showed a latency of a few seconds to select the first box and used consistent routes between feeding boxes, suggesting that the adoption of routes was not modulated by the social environment.

Finally, the preference shown by individuals for a subset of boxes played a central role in the animals' poor performance. Only one individual (M2) has benefited from the use of the route, because his preferred box in most experiments was randomly selected as a reward box for the spatial experiment. This preference for certain boxes is reminiscent of the repetitive use of routes by free-ranging (and semi-captive) owl monkeys, a behavior that can be aimed at reducing the risk of locomotion in little-known (or unknown) places under low-light conditions [16], [17].

Future studies should evaluate the effects of an increase in the experiment's duration and the distance between the feeding boxes on the owl monkeys' learning. The use of sounds produced by insects preyed upon by owl monkeys as auditory cues, the use of insects or other food items as rewards, and the supply of a greater variation in the amount of food among reward boxes are examples of changes in the experimental protocol that may help identify the most salient environmental cues during nocturnal foraging of the owl monkeys.

## Supporting Information

S1 Table **Composition of study groups and enclosure dimensions.**
(DOCX)

S1 Checklist **Completed “The ARRIVE Guidelines Checklist” for reporting animal data in this manuscript.**
(PDF)
