# Supplementary material for: Owl Monkeys (Aotus nigriceps and A. infulatus) Follow Routes Instead of Food-Related Cues during Foraging in Captivity
Source: PLoS One. 2014 Dec 17;9(12):e115188. doi: 10.1371/journal.pone.0115188 (PMC4269449; doi:10.1371/journal.pone.0115188)
Supplement: S1 Table — Composition of study groups and enclosure dimensions. (DOCX) [file pone.0115188.s001.docx]

Table S1: Composition of study groups and enclosure dimensions.

|  |  |  |  |  |
| --- | --- | --- | --- | --- |
| \| Group \| Individual \| Species \| Enclosure dimensions (m)  (width x length x height) \| Area (m^3^) \| \| --- \| --- \| --- \| --- \| --- \| \| 1 \| F1 - Female \| *A. nigriceps* \| 4.50 x 5.10 x 3.15 \| 72.3 \| \| M1 - Male \| *A. nigriceps* \|  \|  \| \|  \|  \|  \|  \|  \| \| 2 \| F2 - Female \| *A. nigriceps* \| 4.50 x 5.10 x 3.15 \| 72.3 \| \| M2 - Male \| *A. infulatus* \|  \|  \| \|  \|  \|  \|  \|  \| \| 3 \| M3 - Male \| *A. infulatus* \| 2.80x 5.60 x 2.90 \| 45.5 \| \|  \|  \|  \|  \|  \| \| 4 \| F4 - Female \| *A. infulatus* \| 2.80 x 5.50 x 2.70 \| 41.6 \| \| M4 - Male \| *A. infulatus* \|  \|  \| \|  \|  \|  \|  \|  \| \| 5 \| F51 - Female \| *A. nigriceps* \| 5.30 x 7.60 x 2.60 \| 104.7 \| \| F52 - Female \| *A. nigriceps* \|  \|  \| \| M5 - Male \| *A. nigriceps* \|  \|  \| |  |  |  |  |
